# Supplementary material for: Deferasirox-Dependent Iron Chelation Enhances Mitochondrial Dysfunction and Restores p53 Signaling by Stabilization of p53 Family Members in Leukemic Cells
Source: Int J Mol Sci. 2020 Oct 16;21(20):7674. doi: 10.3390/ijms21207674 (PMC7589297; doi:10.3390/ijms21207674)
Supplement: Supplementary file 1 [file ijms-21-07674-s001.pdf]

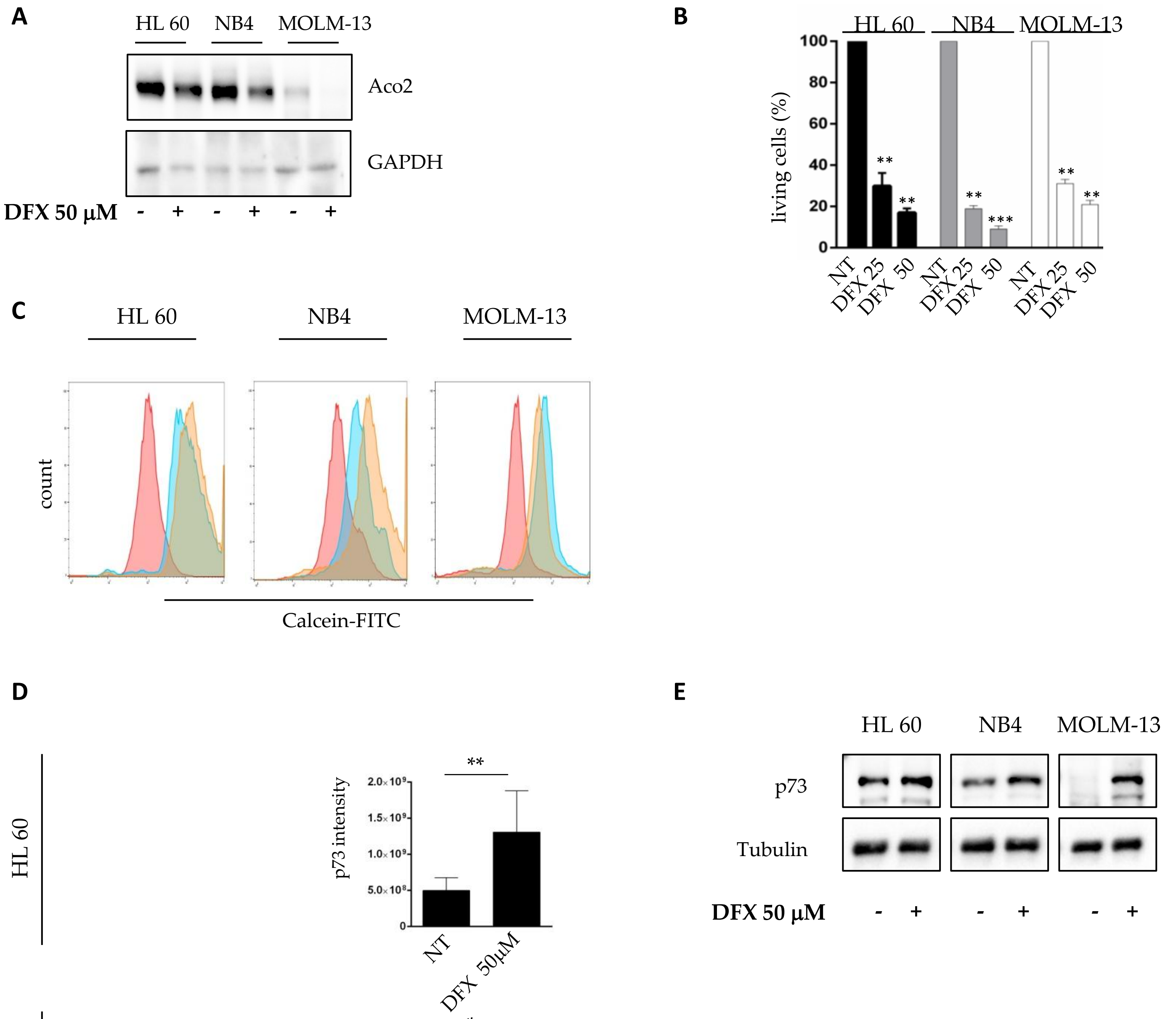

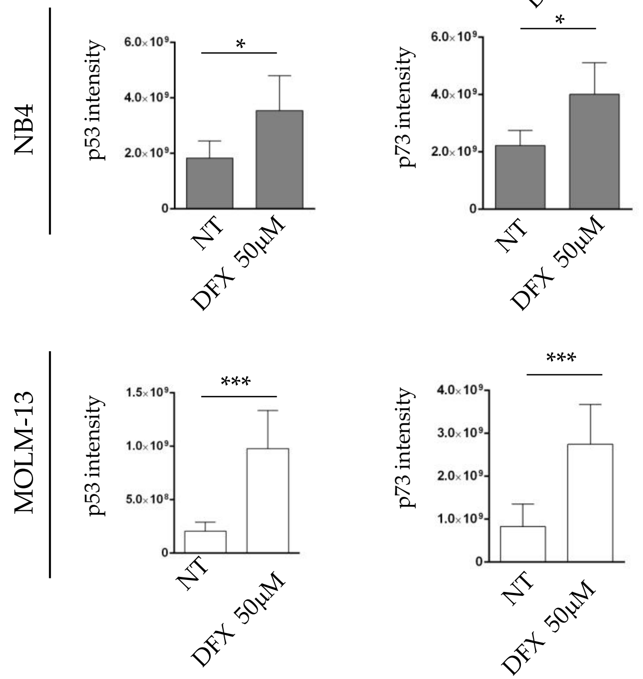

Figure Supplementary S1: Deferasirox reduces mitochondrial activity and leukemia cell lines' viability by increasing p53 and p73 protein levels

A) Aconitase 2 was analyzed by western blot in HL60, NB4 and MOLM-13 after treatment with 50 µM of DFX. B) HL60, NB4 and MOLM-13 living cells were counted at FACS after DFX treatment. C) Istograms overlay of FITC-calcein signal after 48 hours of DFX treatment. LIP level was inversely proportional to measured fluorescence intensity. D) p53 and p73 immunofluorescence signals intensity was quantified by the Java (Image J) program E) p73 was analyzed by Western Blot in HL60, NB4 and MOLM-13 after treatment with 50 µM of DFX.
Abbreviations: -: not treated, +: DFX 50 µM, NT: not treated, * $p \leq 0.05$, ** $p \leq 0.01$.

A

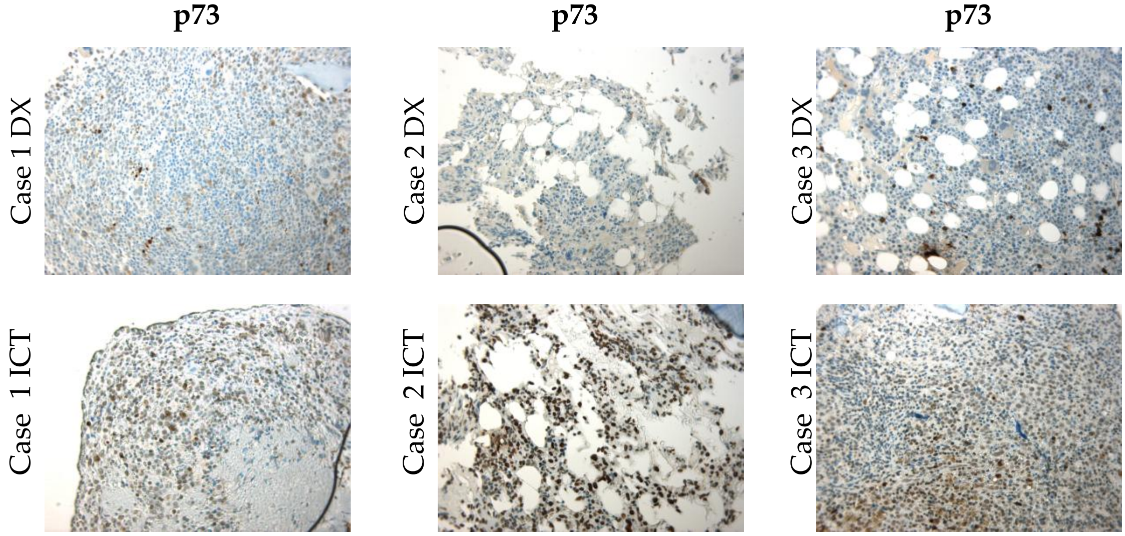

B

Cases	Samples	SCORE p73
**Case 1**	Sample 1-DX	40%
	Sample 1-DFX	70%
**Case 2**	Sample 2-DX	<1%
	Sample 2-DFX	90%
**Case 3**	Sample 3-DX	10%
	Sample 3-DFX	20%
**Case 4**	Sample 4-DX	<1%
	Sample 4-DFX	10%
**Case 5**	Sample 5-DX	90%
	Sample 5-DFX	90%

C

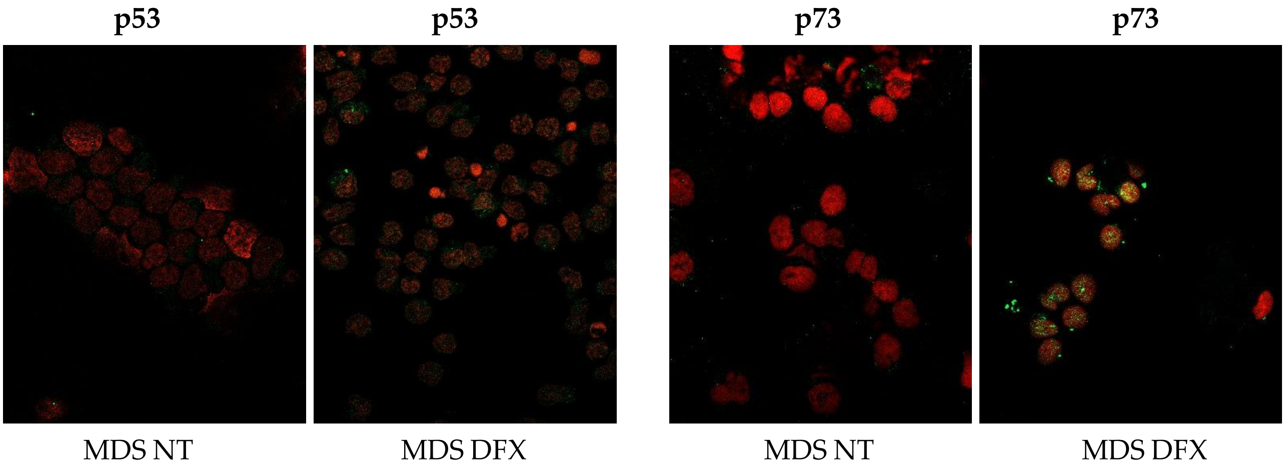

Figure Supplementary S2: Iron chelation treatment induces a significant p73 increase in MDS patients' cells.
A) p73 Immunohistochemistry on MDS bone marrow samples derived from 3 different patients at diagnosis (DX) and within one year of iron chelation treatment (ICT) (10x magnification). B) Table illustrating MDS samples used for IHC analysis and p73 scores. Slides were scored independently by 2 pathologists. C) Immunofluorescence of p53 and p73 in one MDS sample after DFX treatment. The green signal corresponds to p53 or p73 while the red propidium is used to detect nuclei (63x magnification).
Abbreviations: DX: diagnosis, ICT: iron chelation treatment.
